# Sperm quality impairment in males of couples with pregnancy loss is correlated with sexual dysfunction: a cross-sectional study

**DOI:** 10.1186/s12958-023-01067-9

**Published:** 2023-01-28

**Authors:** Xiaowei Yu, Songling Zhang, Xiao Yuan Zhang, Qun Wang

**Affiliations:** 1 Department of Reproductive Medicine, Department of Prenatal Diagnosis, The First Hospital of Jilin University, Changchun, Jilin China; 2 Department of Obstetrics and Gynecology, The First Hospital of Jilin University, Changchun, Jilin China

**Keywords:** Pregnancy loss, Sexual dysfunction, Sperm quality, Anxiety

## Abstract

**Background:**

Erectile dysfunction is a common problem in males of couples experiencing pregnancy loss. Erectile dysfunction in males with couple infertile has been extensively investigated and found to be closely linked with semen quality impairment and psychological distress, but it is less clear if this relation exists in males of couples experiencing pregnancy loss.

**Method:**

A cross-sectional analysis of 437 men who attended our outpatient clinic between June 2021 and October 2021 for couple pregnancy loss. All subjects underwent a complete physical examination, palpation, inspection of the male genitalia, and semen analysis. Validated assessment tools for erectile dysfunction (the International Index of Sexual Function5 -IIEF-5) and anxiety (the seven-item Generalized Anxiety Disorder Scale- GAD-7) were used.

**Results:**

Among 437 men of couples with pregnancy loss, we found several relevant sperm parameters confirmed a significant correlation between IIEF-5 scores and sperm parameters, including: sperm progressive motility (*r* = 0.1627, *p* = 0.001), sperm normal morphology (*r* = 0.1373, *p* = 0.004) and sperm DNA fragmentation (*r* =—0.1248, *p* = 0.009). Males with an IIEF-5 scores range between 5–11 presented the worst results in terms of sperm progressive motility (*p* = 0.002), normal morphology (*p* = 0.001), and SDF levels (*p* = 0.003). GAD-7 score, as well as anxiety level, was significantly higher in those males with an IIEF-5 score between 5 and 11 (*p* = 0.000).

**Conclusion:**

Although current evidence does not demonstrate the importance of spermatozoa in the etiology of pregnancy loss, significant correlations have been observed between impaired sperm quality and low IIEF-5 scores. Also, anxiety is more likely to occur in males with sexual dysfunction.

**Supplementary Information:**

The online version contains supplementary material available at 10.1186/s12958-023-01067-9.

## Introduction

Pregnancy loss (PL) is defined as the spontaneous end of a pregnancy before 24 weeks of gestation, and it is estimated that one-seventh of pregnancies are lost. Recurrent pregnancy loss (RPL) is the failure of two or more pregnancies, which includes embryonic and fetal losses [1]. Importantly, the causes of PL are often unknown; hence, it is not always possible to identify the cause in couple PL [1].

Sperm abnormalities in males may contribute to PL, a phenomenon which is getting more attention in research [2]. In particular, sperm DNA fragmentation (SDF) may be involved in couple PL [3, 4]. Regrettably, there are no clear guidelines for recommending SDF screening. The physician may also not recommend routine or additional sperm testing in the male partners of couples with PL for the etiological screening. In clinical practice, it is recommended for females to be examined first, followed by the males. Screening for karyotype abnormalities in males is widely recommended for couples with PL. The cause of recurring PL is unknown in about half of cases [1]. In most cases, men of PL couple prefer do a semen examination before planning another pregnancy. Male partners frequently blame themselves for the spermatozoa disorders which are thought to cause unfavorable pregnancy outcomes, although these concerns are rarely based on scientific evidence. Great efforts are made toward achieving normal spermatozoa in preparation for pregnancy.

Sperm examination is a pivotal measurement of fertility in couple infertility cases. More importantly, there is a strong correlation between impaired semen quality and sexual dysfunction in the infertile population, with sexual dysfunction tending to be more severe the lower the sperm quality [5, 6]. Many males of PL couples also experience a high level of psychological distress and emotional burden, and sexual dysfunction is increased among them [7–9].

According to the current evidence, spermatozoa abnormalities are not necessarily a major contributor to couple PL [2, 3]. In our previous studies, we showed that erectile dysfunction (ED) is highly prevalent, existing in 30.5% of males with couple PL [10]. It is worth investigating whether impaired sperm quality is related to male sexual dysfunction in males with couple PL. This study utilized survey data from a cohort of males with couple PL from the andrology clinic, attempting to evaluate the relationship between semen quality and male sexual function. Understanding the link between sexual dysfunction and semen quality can help to optimize individual treatments.

## Methods

### Ethics statement

We conducted this study in compliance with the Helsinki declaration and its amendments. The study was approved by the Ethics Committee of the First Hospital of Jilin University (21K064-001). All participants signed an informed consent form. The study is a registered clinical trial in ClinicalTrials.gov and the identifier is NCT04941690).

### Human subject study

This study was a cross-sectional cohort study on a group of 437 male partners in couples with PL that were assessed at a single Reproductive Center at the First Hospital of Jilin University between June 2021 and October 2021. Couples with PL in this study are defined as those who experienced one or more spontaneous ends of a pregnancy before 24 weeks of gestation: embryonic, fetal, and biochemical pregnancy losses were included. Of note, ectopic and molar pregnancies were excluded in the present study. Male patients enrolled were: i. greater than or equal to 20 years old and seeking medical care for couple PL. ii. Men living together with wives and having regular intercourse during the study period; iii. Members of couples planning to try to conceive; iv. Men who voluntarily came to our andrology clinic to seek medical help. The exclusion criteria were: i. Men with a previous physician diagnosis of severe cardiovascular diseases, hypogonadism, or brain strokes;ii. Men separated from their wives or without regular sexual intercourse, at least once a month (for example, in cases where female surgical treatment or vaginal operation prevented sexual intercourse); iii. Men with psychopathological conditions or who were receiving medications that may affect sexual function (such as phosphodiesterase 5 inhibitors, testosterone, and selective serotonin reuptake inhibitors). Since most of the males attended the clinic by themselves, we were unable to evaluate the fertility and sexual function of their female partners.

### Self-reported questionnaires

All participants completed a web-based questionnaire that included comprehensive demographic information, as well as the five-item version of the International Index of Erectile Function (IIEF-5) for diagnosis of ED; anxiety was assessed by the seven-item Generalized Anxiety Disorder (GAD-7) Scale. Timed intercourse is defined in this study as when more than 70% of sexual intercourse in a month is concentrated around the time of ovulation. The predicted ovulation day is based on various ovulation prediction methods, such as calendar charting, tracking basal body temperature, cervical secretion investigation, and urinary hormone measurement. A GAD-7 with a score of 5 or more is considered an anxiety state.

### Semen analysis and physical examination

After sexual abstinence of three to seven days, samples of semen were collected in sterile containers by masturbation. Sperm quality was defined according to World Health Organization guidelines [11]. The mean of SDF was determiend by TUNEL assay; DNA fragmentation was assessed 1 h after ejaculation to avoid iatrogenic DNA damage. Different cutoff values of SDF were used to discriminate between normal and high-SDF specimens; most studies used 30% or greater as the cutoff value for high-SDF [12]. Testicular parenchyma or varicocele were palpated during physical examinations. A Prader orchidometer was used to assess testes volume, and a solo physician performed all physical examinations.

### Data analysis

A mean ± standard deviation (SD) was calculated for normally distributed data, a median (quartile) for non-normally distributed data, and a percentage for categorical data. Analysis of variance (ANOVA) or Kruskal–Wallis tests were used for comparisons of more than two groups for continuous parameters, and categorical variables were compared using the Pearson χ2 test. Correlations were analyzed using Spearman’s correlation test and regression analysis. Analysis of covariance (ANCOVA) was used to investigate the association between changes in sperm quality and sexual dysfunction, adjusted for potential confounding factors (i.e. variables statistically significant in the univariate analysis). *P*-values < 0.05 were considered statistically significant. Data entry and analysis were performed using the IBM SPSS Statistics for Windows, Version 22.0 (Armonk, NY: IBM Corp) software package.

## Results

### Participant characteristics

A total of 437 male partners in couples with PL met the inclusion criteria for the study. Table 1 summarizes the patient characteristics. When compared with participants of normal sexual function, patients with ED tended to be older (32.09 ± 4.19 vs. 34.11 ± 4.55; *P* = 0.000), have more incidences of PL (*P* = 0.001), employ timed intercourse more frequently (*P* = 0.000), and have higher levels of anxiety (*P* = 0.000). No group differences were evident in the remaining demographic variables. (See Table 1).

**Table 1:** Sociodemographic and clinical characteristics of the participants

Demographics and clinical parameters	Males with normal sexual function (*N* = 318)	Males with erectile dysfunction (*N* = 119)	P value
Age (years)	32.09 ± 4.19	34.11 ± 4.55	**0.000**
Body-mass index (kg/m^2^)	25.49 ± 3.40	25.91 ± 3.53	0.257
Current smokers (%)	37.7	44.5	0.196
Current alcohol consumption (≥ 4 drinks/week) (%)	18.5	19.3	0.853
Education level (%)			0.679
No higher than high school	19.2	20.0	
High school	23.3	26.9	
University and above	57.5	57.1	
Monthly household income			0.349
~ 5000	24.5	22.7	
5000–7000	60.0	47.9	
7000 ~	24.5	29.4	
Night shifts (times/week) (%)			0.215
1–2	11.6	15.9	
3–4	3.1	1.7	
> 4	1.2	0.8	
Number of pregnancy losss (%)			**0.001**
1	44.0	30.2	
2	41.5	43.7	
≥ 3	14.5	26.1	
Timed intercourse (%)	11.3	33.6	**0.000**
GAD-7 scores	2.75 ± 2.08	4.50 ± 3.13	**0.000**
Anxiety state (%)	18.2	46.2	**0.000**
Mean testis volume (Prader) (ml)	13.44 ± 2.73	13.37 ± 2.81	0.803
Clinical varicocele (Palpation) (%)	8.5	13.4	0.121
Symptoms of Prostatitis (%)	8.5	11.8	0.296

Significant results are shown in bold (*p* < 0.05, paired t test). Group A; males with ED; Group B: males with normal sexual function

### Erectile dysfunction and sperm quality

**Fig. 1:**
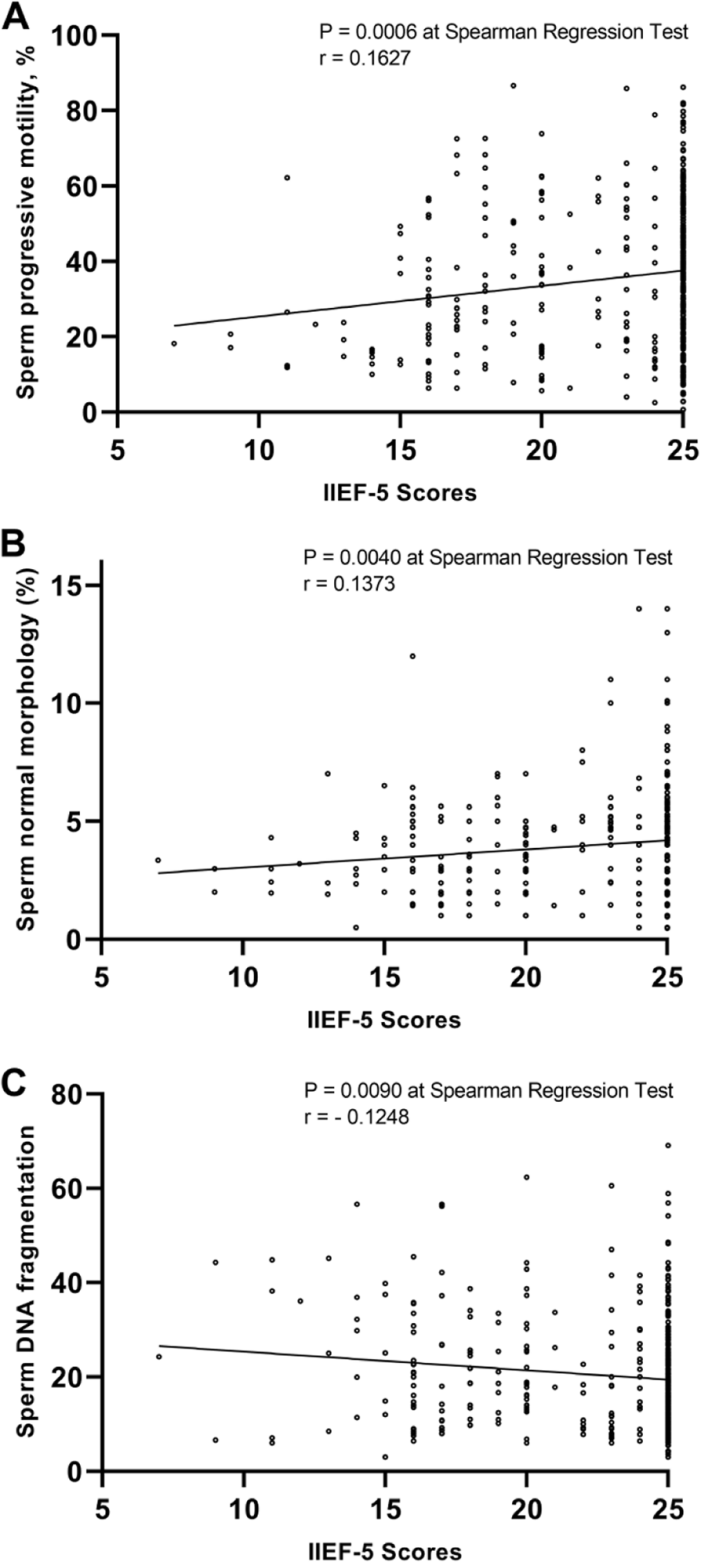
Relationship between IIEF-5 scores and sperm quality (**A**: sperm progressive motility, **B**: sperm normal morphology and **C**: sperm DNA fragmentation)

Figure 1 shows that there are significant positive correlations between sperm progressive motility (*r* = 0.1627, *p* = 0.001), sperm normal morphology (*r* = 0.1373, *p* = 0.004) and IIEF-5 scores. Significant negative correlations were also seen between SDF (*r* =—0.1248, *p* = 0.009) and IIEF-5 scores. (See Fig. 1) There were no such correlations in other sperm parameters, such as semen volume, sperm concentration, and total sperm number. (See Supplementary Fig. 1) The analysis was performed after adjusting for possible confounding factors related to age.

**Fig. 2:**
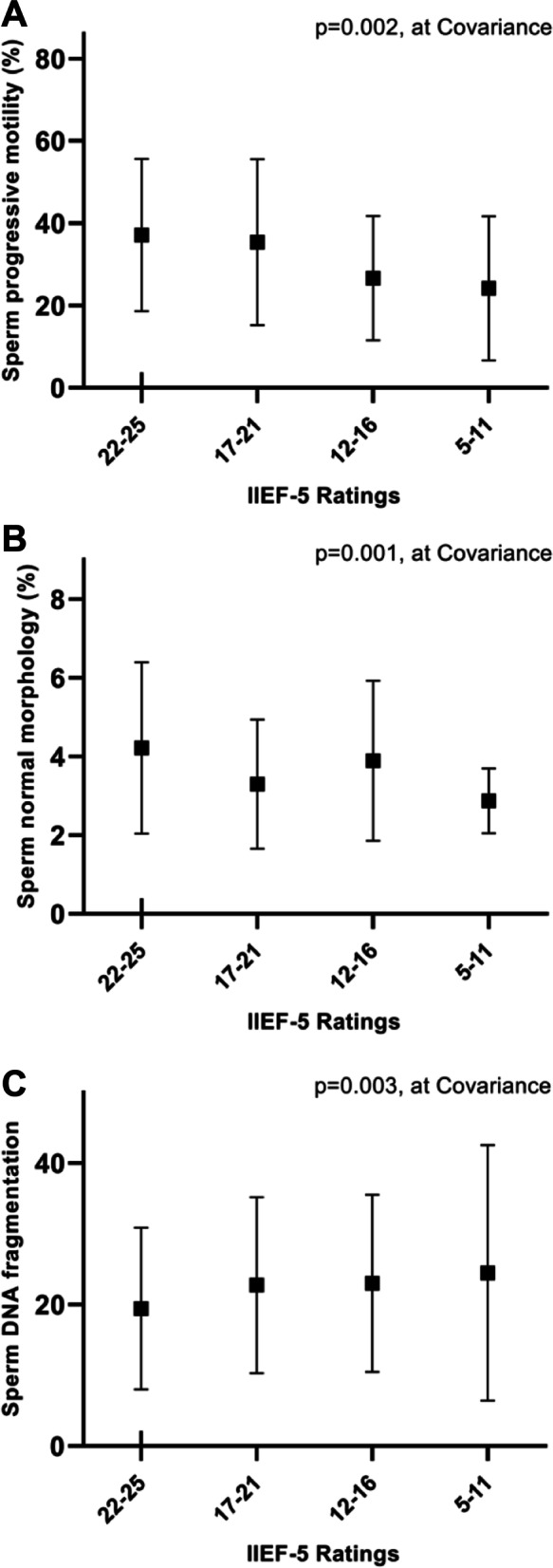
Comparison among groups of men for the severity of erectile dysfunction and sperm quality (**A**: sperm progressive motility, **B**: sperm normal morphology and **C**: sperm DNA fragmentation). Comparisons were performed after adjustment for age. Graphs for each group considered show the mean and standard deviation of the parameters evaluated

According to the severity of ED, IIEF-5 scores can be classified as 22–25 (without ED), 17–21 (mild), 12–16 (mild to moderate), or 5–11 (moderate-severe). Males with IIEF-5 score range between 5–11 (moderate-severe ED) presented the worst results in terms of sperm progressive motility (*p* = 0.002), normal morphology (*p* = 0.001), and SDF levels (*p* = 0.003). (See Fig. 2) Other sperm indices (semen volume, sperm concentration, and total sperm number) were not significantly different between groups. (See Supplementary Fig. 2).

### Anxiety

**Fig. 3:**
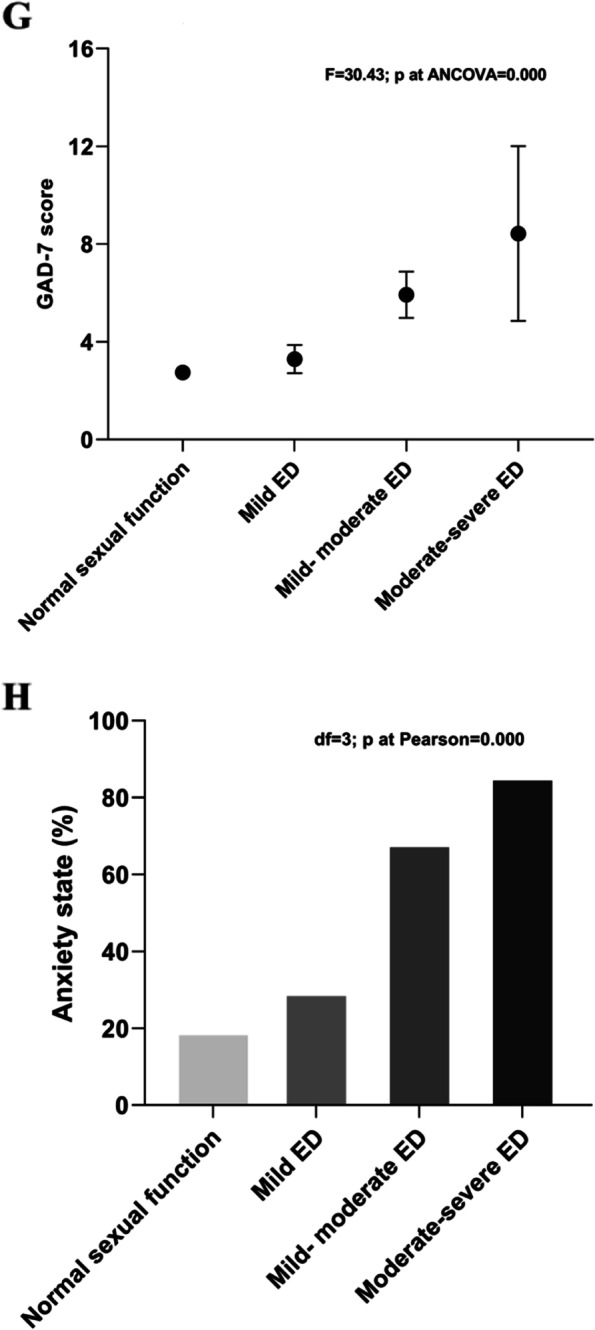
Comparison among groups of men for the severity of erectile dysfunction (ED) and anxiety. Comparison among groups for: GAD-7 scores (panel **G**); Anxiety state (%) (panel **H**). Comparisons were performed after adjustment for age. Graphs for each group considered show the mean and standard deviation (panel **G**) or percentage (panel **H**) of the parameters evaluated

From the analysis of covariance in the anxiety score, scores in the normal sexual function group (IIEF-5 score: 22–25) were significantly lower than in the ED group (IIEF-5 score ≤ 22) (2.75 ± 2.08 vs. 4.50 ± 3.13; *P* = 0.000). The GAD-7 score increased with ED severity (See Fig. 3G). The proportion of patients who reported anxiety also increased with increase in severity of the ED, more than half of the study participants had mild to moderate ED (IIEF-5 score: 12–16, 68.3%) or moderate to severe ED (IIEF-5 score: 5–11, 85.7%) with self-reported anxiety. (See Fig. 3F).

### Timed intercourse

**Fig. 4:**
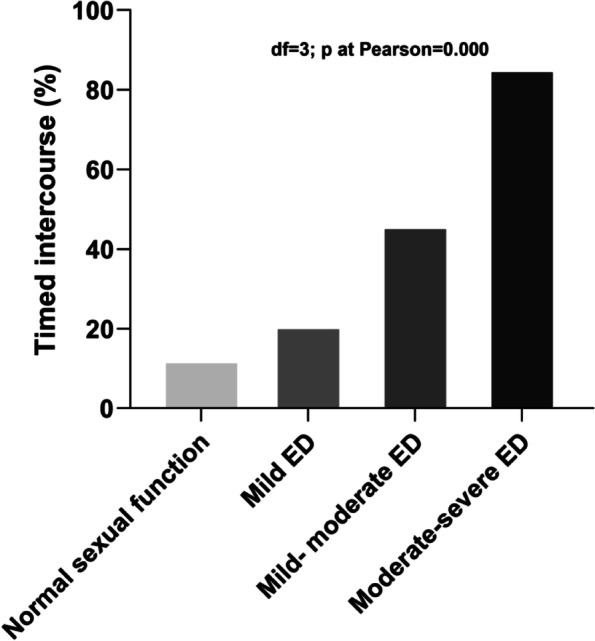
Comparison among groups of men for the severity of erectile dysfunction (ED) and timed intercourse (%)

Men with ED were more inclined to organize their sexual lives according to the fertility window to maximize the chance of conception, compared to males with normal sexual function (33.6% vs. 11.3%; *P* = 0.000). As the severity of ED increased, male participants were more likely to prefer timed intercourse over regular sexual intercourse. Sexual intercourse during ovulation was common in half or more of the participants with mild to moderate ED (IIEF-5 score: 12–16, 46.3%) or moderate to severe ED (IIEF-5 score: 5–11, 85.7%). (See Fig. 4).

## Discussion

To our knowledge, the present study is the first to investigate the association between ED, defined by traditional IIEF-5 scores and sperm quality, and psychological distress (anxiety) among male partners of PL couples. In males of PL couples, with the decrease of sperm progressive motility/normal morphology and elevated SDF levels, IIEF-5 scores show a significant downward trend. Similarly, when differentiating the severity of ED based on the value of IIEF-5 scores, males with lowest IIEF-5 scores range (5–11) also presented the worst results in terms of sperm progressive motility/normal morphology and SDF levels. GAD-7 score, as well as anxiety levels, were significantly elevated in those males with IIEF-5 score between 5 and 11 (moderate-severe ED), and they were more inclined to arrange their sexual lives according to the “fertile window.”

Established causes of PL classically include uterine anomalies, hormonal and metabolic disorders (hypothyroidism and diabetes), infections (chronic endometritis), autoimmune abnormalities (antiphospholipid antibody syndrome, thrombophilias), and abnormal chromosomes (particularly translocations, in either partner). Embryonic aneuploidy as the most common cause of sporadic PL, primarily derived from oocyte [13]. In clinical practice, screening only for karyotype abnormalities in males is widely recommended among couples with PL. Despite thorough evaluation, at least 50% of cases of RPL cannot be attributed to a specific etiology. PL couples are repeatedly told that in the absence of treatment, up to 60% to 70% of couples with unexplained RPL will have success with their next pregnancy [14]. However, the absence of identifiable causes in either partner is difficult for patients to accept, as pregnancy loss is a painful experience [15]. At present, the etiology and pathogenesis of PL have been and continue to be intensely investigated, and there has been increased appreciation for the role of sperm in PL. Many investigators have found sperm factors may be associated with PL. Elevated levels of SDF has been linked to early gestation pregnancy loss (prior to 20 weeks) [3] and high proportions of sperm aneuploidy may present even among patients with a normal male karyotype (46, XY) [16]. Ejaculate sperm contain approximately 0.6% aneuploid chromosomes. This incidence increases to 6% in severe oligospermia with disordered spermatogenesis and up to 14% in nonobstructive azoospermia [2]. Regrettably, there are no clear guidelines for recommending routine sperm test for the etiological screening of couple PL. These couples are without a universal treatment recommendation, and many couples express fear over potential complications in the next pregnancy and are reluctant to conceive again.

Although the magnitude of the correlation between sperm quality impairment and couple PL is modest, after all, half of the embryo's genetic material is contributed by the male partner. Men with couple PL often feel they need to do pre-pregnancy semen examinations before planning a pregnancy, and if these examination show abnormalities (asthenozoospermia, teratozoopermia, oligospermia), many couples are reluctant to conceive again because of the potential pregnancy complications. In clinical work, nearly all teratozoopermia patients expressed that teratozoopermia is a serious concern for them, and worried about the involvement of teratozoopermiain sperm-egg binding and fertilization. These concerns are rarely based on scientific evidence, but great efforts are made to improve the sperm quality in these patients, who look to achieve normal spermatozoa in preparation for pregnancy.

Couples trying to conceive were recommended to engage in regular intercourse at least two to three times a week [17]. In the current study, couples who had experienced PL were more inclined to arrange their sexual lives according to the “fertile window.” Timed intercourse was seen in 33.6% of men with ED in PL couples, whereas in the normal sexual function group this proportion is only 11.3%. Timed intercourse may not ultimately be beneficial in achieving pregnancy, but instead increase stress related to timed intercourse as well as the risk of sexual dysfunction [18, 19]. Ejaculation frequency is also an important factor that influences semen parameters. It is widely admitted that prolonged sexual abstinence may be beneficial for semen volume and sperm concentration; however, lack of ejaculation also displays adverse consequences on sperm motility, viability, and SDF [20]. Increasing the number of ejaculations may be a way of improving sperm parameters and SDF, and therefore pregnancy outcomes [21–23]. Recurrent ejaculations have been proposed as a way to improve sperm DNA quality and reproductive success [22–25].

Semen quality is regulated by sex hormones, and the hypothalamic–pituitary–gonadal axis can be altered by emotional disorders such as anxiety [26, 27]. Anxiety was associated with lower sperm parameters and higher SDF in males [28]. Infertile men showed increased somatized anxiety closely associated with reduced erectile function, sexual desire, and ejaculatory latency [6]. This study showed that male partners with sexual dysfunction in PL couples are more likely to have emotional disorders than males who have normal erectile function, and mild anxiety was more common in males with ED.

This study has limitations that should be noted. First, we were unable to include important variables such as sex hormone profiles, sex hormones not only play an important role in spermatogenesis and sperm maturation [29] but also in the pathogenesis of sexual dysfunction [30]. Thus, a strong bidirectional association between sex hormones and sperm quality/ sexual function may exist. Second, male sexual dysfunction can also be aggravated by the coexistence of sexual dysfunction in the female partner [5]. Unfortunately, in conservative cultures like China, we do not have access to female sexual function data. In addition, the analysis was cross-sectional and hospital-based, which raises the possibility of selection bias, and there may have been a selection bias regarding the control group. Thus, results should be interpreted with caution. Research on large prospective studies is still needed to verify our findings.

In summary, although there is insufficient evidence from the current studies to support the importance of spermatozoa in the etiology of PL, a significant correlation was observed between poor sperm quality and a lower IIEF-5 score. Males with the lowest IIEF-5 scores (5–11) showed the worst results in terms of sperm motility, normal morphology, and sperm SDF levels. The GAD-7 score, as well as anxiety levels, were significantly increased.

## Supplementary Information

**Additional file 1.**

## Data Availability

The datasets used and/or analyzed during the current study are available from the corresponding author on reasonable request.

## Abbreviations

PL: Pregnancy loss
RPL: Recurrent pregnancy loss
SDF: Sperm DNA fragmentation
ED: Erectile dysfunction
IIEF-5: Five-item version of the International Index of Erectile Function
GAD-7: Seven-item Generalized Anxiety Disorder
SD: Standard deviation. ANOVA:analysis of variance
ANCOVA: Analysis of covariance
