# Supplementary material for: Sperm quality impairment in males of couples with pregnancy loss is correlated with sexual dysfunction: a cross-sectional study
Source: Reprod Biol Endocrinol. 2023 Jan 28;21:11. doi: 10.1186/s12958-023-01067-9 (PMC9883875; doi:10.1186/s12958-023-01067-9)
Supplement: Supplementary file 1 — Additional file 1. [file 12958_2023_1067_MOESM1_ESM.docx]

**Supplementary Figure 1** Relationship between IIEF-5 scores and sperm quality (A: semen volume, B: sperm concentration and C: total sperm number)


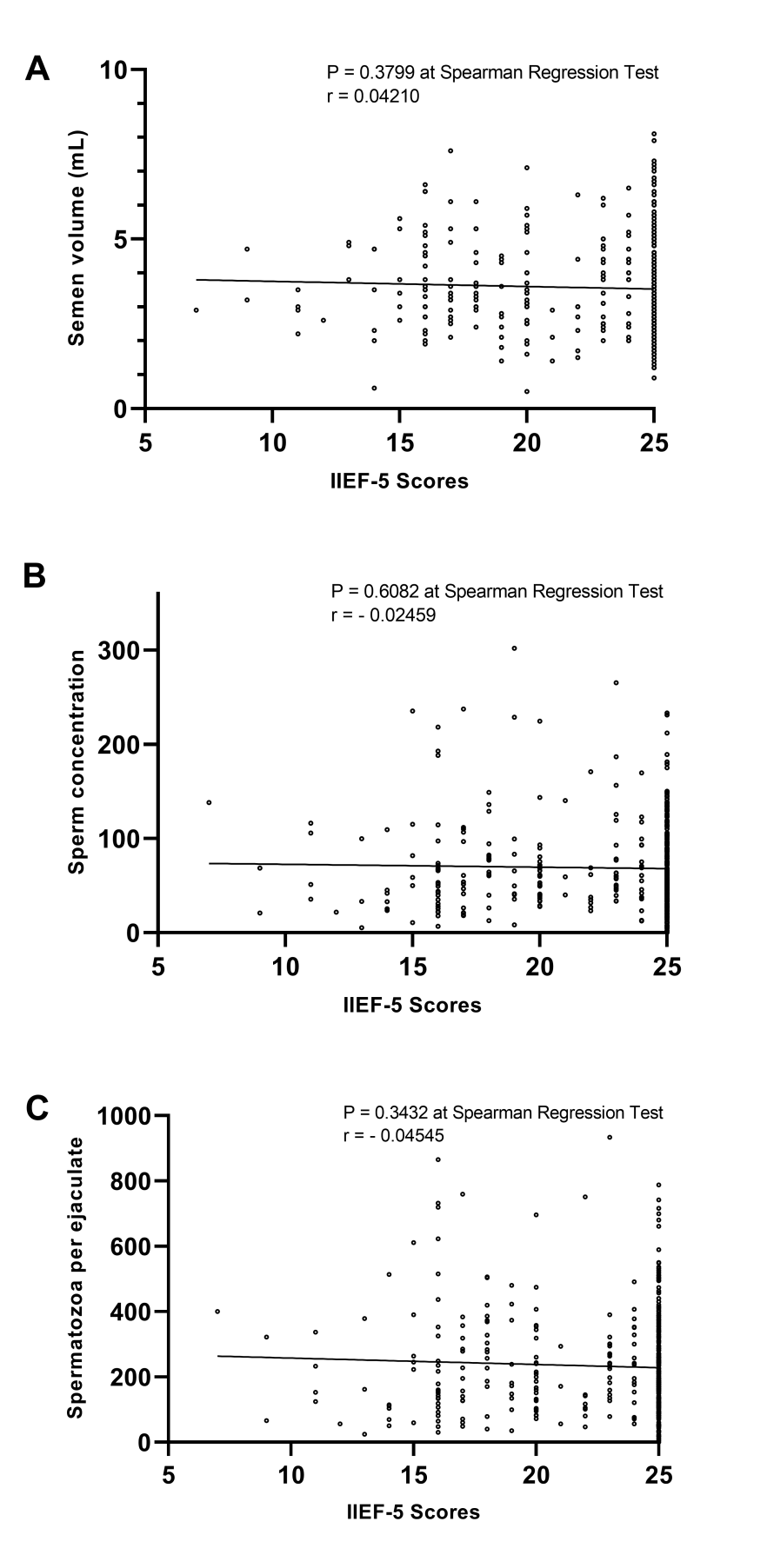


**Supplementary Figure 2** Comparison among groups of men for the severity of erectile dysfunction and sperm quality (A: semen volume, B: sperm concentration and C: total sperm number)


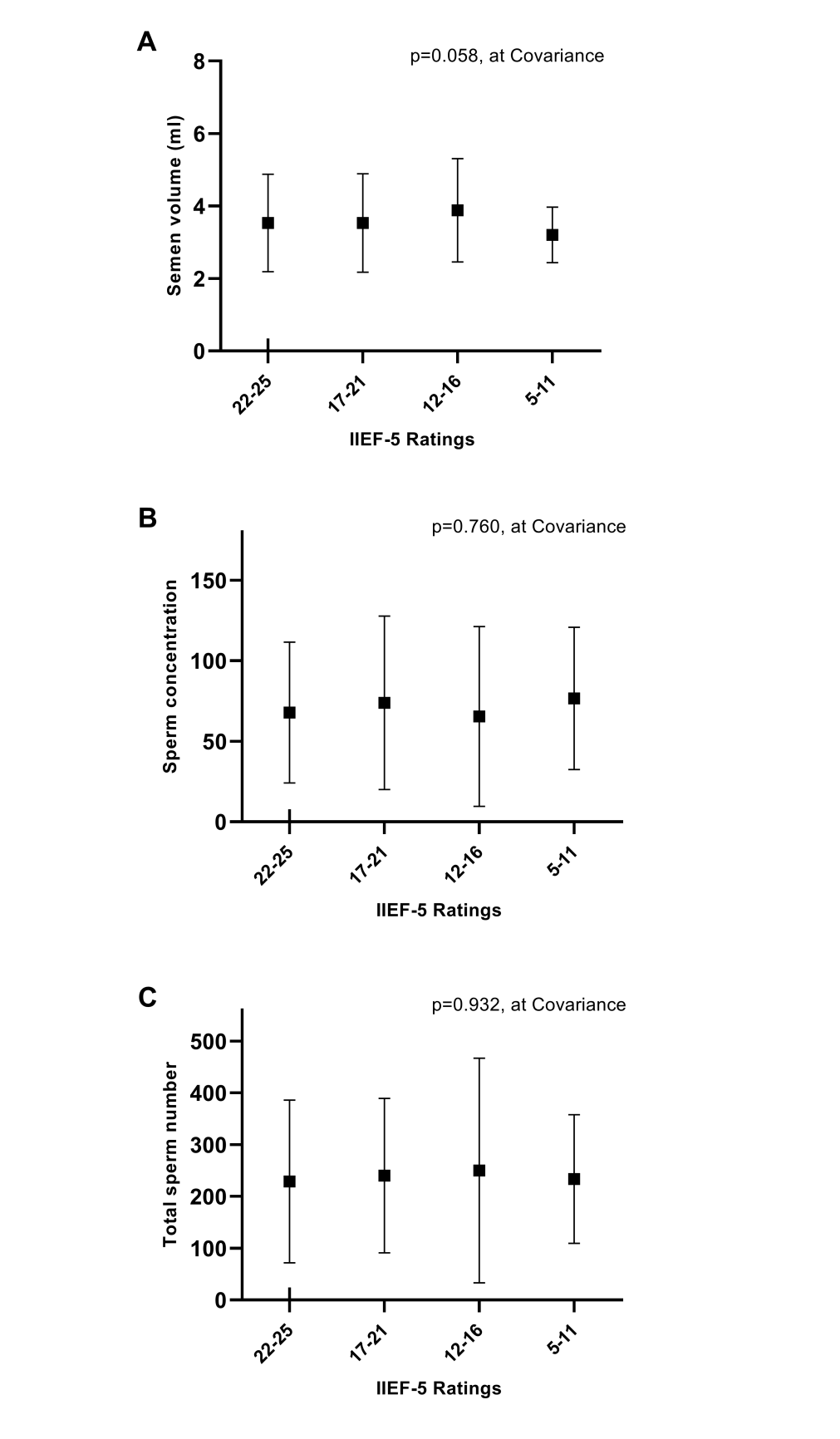


Comparisons were performed after adjustment for age. Graphs for each group considered show the mean and standard deviation of the parameters evaluated.
